# Overexpression of LncRNA BM466146 Predicts Better Prognosis of Breast Cancer

**DOI:** 10.3389/fonc.2020.628757

**Published:** 2021-01-29

**Authors:** Yunxiang Zhang, Xiaotong Dong, Yang Wang, Liquan Wang, Guiyan Han, Lvcheng Jin, Yanping Fan, Guodong Xu, Dawei Yuan, Jie Zheng, Xiangyu Guo, Peng Gao

**Affiliations:** ^1^Key Laboratory for Experimental Teratology of the Ministry of Education, Department of Pathology, School of Basic Medical Sciences, Shandong University, Jinan, China; ^2^Pathology Department, First Affiliated Hospital of Weifang Medical University, (Weifang People’s Hospital), Weifang, China; ^3^Breast Surgery Department, First Affiliated Hospital of Weifang Medical University, (Weifang People’s Hospital), Weifang, China; ^4^Precision Medicine Department, Geneis Beijing Co., Ltd., Beijing, China; ^5^Department of Diagnostic Pathology, Weifang Medical University, Weifang, China; ^6^Department of Pathology, Qilu Hospital, Shandong University, Jinan, China

**Keywords:** long non-coding RNA, BM466146, breast cancer, CD8^+^ T cells, prognosis

## Abstract

This study analyzes the expression and clinical significance of long non-coding RNA (lncRNA) BM466146 in breast cancer, and explores the role of BM466146 in immune regulation. The expression of BM466146 in 89 cases of breast cancer and their corresponding non-cancerous breast tissues was detected by quantitative real-time polymerase chain reaction (qRT-PCR). Kaplan-Meier survival analysis was applied to evaluate patient survival. EDU and CCK-8 experiments on breast cancer cells were performed to verify the function of BM466146 *in vitro*. The target genes of BM466146 were screened by informatics analysis to predict associated miRNAs and their corresponding mRNAs, immune genes associated with lncRNAs and chemokines associated with CD8. Immunohistochemistry was used to detect the expression of CD8, Ki-67, and CXCL-13 in the 89 breast cancer tissues. It was found that the expression of lncRNA BM466146 in breast cancer tissues was significantly lower than that in normal breast tissues (*P* < 0.001). In breast cancer, tissues that overexpressed BM466146 exhibited a lower Ki-67 index compared with that of low BM466146 expression (*P* = 0.048). Kaplan-Meier survival analysis showed that breast cancer patients with overexpression of BM466146 had longer overall survival. EDU and CCK8 experiments showed that overexpression of BM466146 inhibited the proliferation of breast cancer cells. The hsa-miR-224-3p is associated with BM466146, and its target gene might be CXCL-13. The positive CD8 cells in the BM466146 overexpression group was higher than that in the low BM466146 expression group (*P*=0.027), and the positive CD8 cells in the CXCL-13 positive group was higher (*P*=0.023) than that of the negative group. Our results indicate that the lncRNA BM466146 has the function of tumor suppressor gene. Overexpression of BM466146 is associated with better prognosis. BM466146 could regulate CXCL-13 by adsorbing hsa-miR-224-3p and inducing CD8^+^ T cells to accumulate in the tumor area which regulate immune response. Therefore, BM466146 could be a prognostic biomarker and a molecular immune target of breast cancer.

## Introduction

Breast cancer is the most common malignant tumor that threatens women’s health. The latest statistics in recent years show that the incidence and mortality of breast cancer remains high globally (1, 2). Chemotherapy is currently the main treatment modality for breast cancer, but drug resistance of tumor limits the effectiveness of treatment (3). Studies have demonstrated that a large number of immune cells, cytokines and growth factors exist in the tumor microenvironment of breast cancer. These factors play important roles in tumor proliferation, migration, and invasion (4). There is evidence that tumor-infiltrating lymphocytes can affect the prognosis and treatment response of ductal carcinoma *in situ* and invasive breast cancers. The tumor immune environment before treatment can be used as an indicator for the prognosis of individual diseases, as well as treatment guide (5). However, the mechanism of immune system related to the occurrence, metastasis, treatment, and prognosis of breast cancer is not clear. Therefore, exploring the pathogenesis of breast cancer and finding targets for breast cancer immunotherapy is the key to prolong the survival of patients and improv their quality of life (6).

Human genomic DNA is composed of 3 billion base pairs, but only <2% of base pairs encode functional proteins, and about 98% of genomic DNA is transcribed into non-coding RNA (7), of which lncRNA is an important component. At present, the role of lncRNA in various cancers such as liver cancer, breast cancer, bladder cancer and other tumors has been reported (8–10). However, the functions and mechanisms of lncRNA in the development of breast cancer have been less studied. In our preliminary microarray analysis on lncRNA, we found that the expression of lncRNA BM466146 in cancer was significantly reduced compared with normal tissue. We therefore explore the biological function and molecular mechanisms of BM466146 in breast cancer, particularly its role in the development and prognosis of breast cancer.

## Materials and Methods

### Specimen Collection

In this study, 89 pairs of fresh breast cancer tissue specimens and matched normal breast tissues (≥5 cm from the tumor) were collected from the hospitalized patients in Weifang People’s Hospital from February 2019 to June 2019. Each half of the tissues was quickly stored in a -80℃ ultra-low temperature refrigerator for qRT-PCR detection, and the other half of the tissues was placed in 4% neutral formaldehyde solution for HE and immunohistochemical staining. The clinical data of all specimens were complete, and two senior pathologists independently read and evaluated the tissues according to the 2019 version of the WHO diagnostic criteria. The study was approved by the Ethics Committee of Weifang People’s Hospital and the participated patients submitted their informed consent.

### Immunohistochemistry

Immunohistochemical staining was used to detect the expression of Ki-67, CD8, CXCL-13 in breast cancer tissues. High temperature and high pressure tissue antigen retrieval method with citrate buffer (PH 9.0) was used to restore the antigens. DAB was used for color development. Neutral gum was used to mount the slides. Positive control was set in each staining. The Ki-67 antibody is a mouse anti-human monoclonal antibody (Ready-to-use antibodies, Fuzhou Maixin, China). The CD8 antibody is a rabbit anti-human monoclonal antibody (Ready-to-use antibodies, Beijing Zhongshan Jinqiao, China). The CXCL-13 antibody is a goat anti-human polyclonal antibody (Ready-to-use antibodies, Fuzhou Maixin, China). A positive result means that there is brownish yellow staining on the nucleus (Ki-67), cell membrane (CD8) or cytoplasm (CXCL-13) of a specific cell in the tissue section, and the number of positive cells is more than 5%.

### RNA Extraction and qRT-PCR

Total RNA of fresh breast cancer and normal breast tissues was extracted using Axygen Total RNA Small Amount Preparation Kit (Corning Life Sciences Co., Ltd., Suzhou, China). UV spectrophotometer (One Drop OD 1000, Nanjing Wuyi Technology Co., Ltd., China) was used to examine the concentration and purity of total RNA by A260/A280 nm ratio. M-MuLV reverse transcriptase (200 U/μl, purchased from New England Biolabs), RNase inhibitor (Takara), and dNTP (Takara) were used to synthesize cDNA. The kit used for qRT-PCR is KAPA SYBR FAST qPCR Kits (purchased from KAPA). GAPDH is selected as the internal reference. The primer sequence is as follows:

BM466146 forward primer 5’-ACCTGACCCATCTACCTTGC-3’,

reversed primer 5’-GTGGTGCTCGCCTGTAATC-3’;

GAPDH forward primer 5’-GCACCGTCAA-GGCTGAGAAC-3’,

reversed primer 5’-TGGTGAAGACGCCAGTGGA-3’.

The PCR was performed using the Applied Biosystems 7500. All tests were performed in triplicates.

### Cell Culture

The Breast cancer MDA-MD-231 cell line was purchased from Procell Life Science & Technology Co., Ltd. (Wuhan, China) and cultured in RPM1640 medium (Solarbio, USA) containing 10% fetal bovine serum (FBS), and cultivated in an incubator with 5%CO_2_ at 37°C.

### Plasmid Construction and Cell Transfection

The pcDNA3.1-BM466146 overexpression plasmid was constructed by Jinan Boshang Biotechnology Co., Ltd. (Jinan, China), PcDNA3.1-BM466146 overexpressed plasmid was transfected into the MDA-MD-231 cells. The pcDNA3.1 vector was used as the control. The cell function experiments were conducted 24 h after transfection.

### CCK-8 Assay

BBoneBot CCK-8 cell proliferation-cytotoxicity detection kit (Shanghai bestbio Biotechnology Co., Ltd., Shanghai, China) was used to detect the proliferation of the cells. MDA-MD-231 cells after 24 h of transfection was seeded at 4 × 10^3^ cells per well in a 96-well plate, with four replicate wells in each group, and the absorbance at 450nm wavelength was measured at 24, 48, 72, and 96 h.

### EDU Assay

BeyoClick EdU-594 cell proliferation detection kit (Shanghai Biyuntian Biotechnology Co., Ltd., Shanghai, China) was used to detect cell proliferation. MDA-MD-231 cells transfected for 24 h was seeded with 1 × 10^4^ cells per well in a 96-well plate, with three replicate wells in each group, and the cell proliferation efficiency was calculated after fluorescent staining.

### Cell Migration and Invasion Assay

Transwell chamber (Corning, Cambridge, MA, USA) was used to detect the effect of overexpression of BM466146 on the migration and invasion of MDA-MD-231 cells. In the migration experiment, the cells in serum-free medium (3 × 10^4^ cells/200 ul) were added to the inner chamber, and the medium containing 10% FBS was added to the outer chamber. After incubating for 24 h, the cells in the inner chamber were wiped out with a cotton swab. The cells that pass through the membrane and adhere to the bottom of the membrane were fixed and then subjected to Giemsa staining. For the invasion experiment, a transwell chamber coated with Matrigel matrix (BD Bioscience) was used for the experiment.

### Bioinformatics

The RNA-seq datasets of breast cancer patients, along with the clinical information, were downloaded from the TCGA database through GDC data transfer tool. The expression level of each gene was generated by analyzing RNA-seq datasets with reference of the Genome Reference Consortium Human genome build 38 (GRCh38). Then, all gene expression data were combined as the gene expression profile for breast cancer. The data were analyzed in combination with the data from human reference genome 19 (hg19) published by the University of California, Santa Cuz (UCSC). The survival curve was drawn based on the data.

The lncRNA and genes that are related to immune functions, and CD8^+^ T cell-related chemokines were searched through The Encyclopedia of RNA Interactomes (ENCORI, http://starbase.sysu.edu.cn/index.php) database. The miRNAs and target genes related to BM466146 were searched through miRNA datasets. Target genes were obtained in the intersection of immune genes related to lncRNA, chemokines related to CD8^+^T cells, and target genes of miRNA.

### Statistical Analysis

Statistical analysis was performed using SPSS Statistics 26 software. Mann-Whitney U rank sum was used to test the significance of statistical differences, Receiver Operating Characteristic (ROC curve) was used to determine the cut off value of lncRNA BM466146 expression. Chi-square test and Fisher exact test were used to analyze the relationship between the expression of lncRNA BM466146, Ki-67 index and CD8. Chi-square test was used to analyze the relationship between CXCL-13 and CD8. Spearman correlation test was used for correlation analysis. Student’s t-test was used for the difference between the experimental group and the control group. *P* value<0.05 is regarded as statistically significant.

## Result

### Expression of lncRNA BM466146 Is Significantly Lower in Breast Cancer Compared With Non-Cancerous Tissues

**Figure 1:**
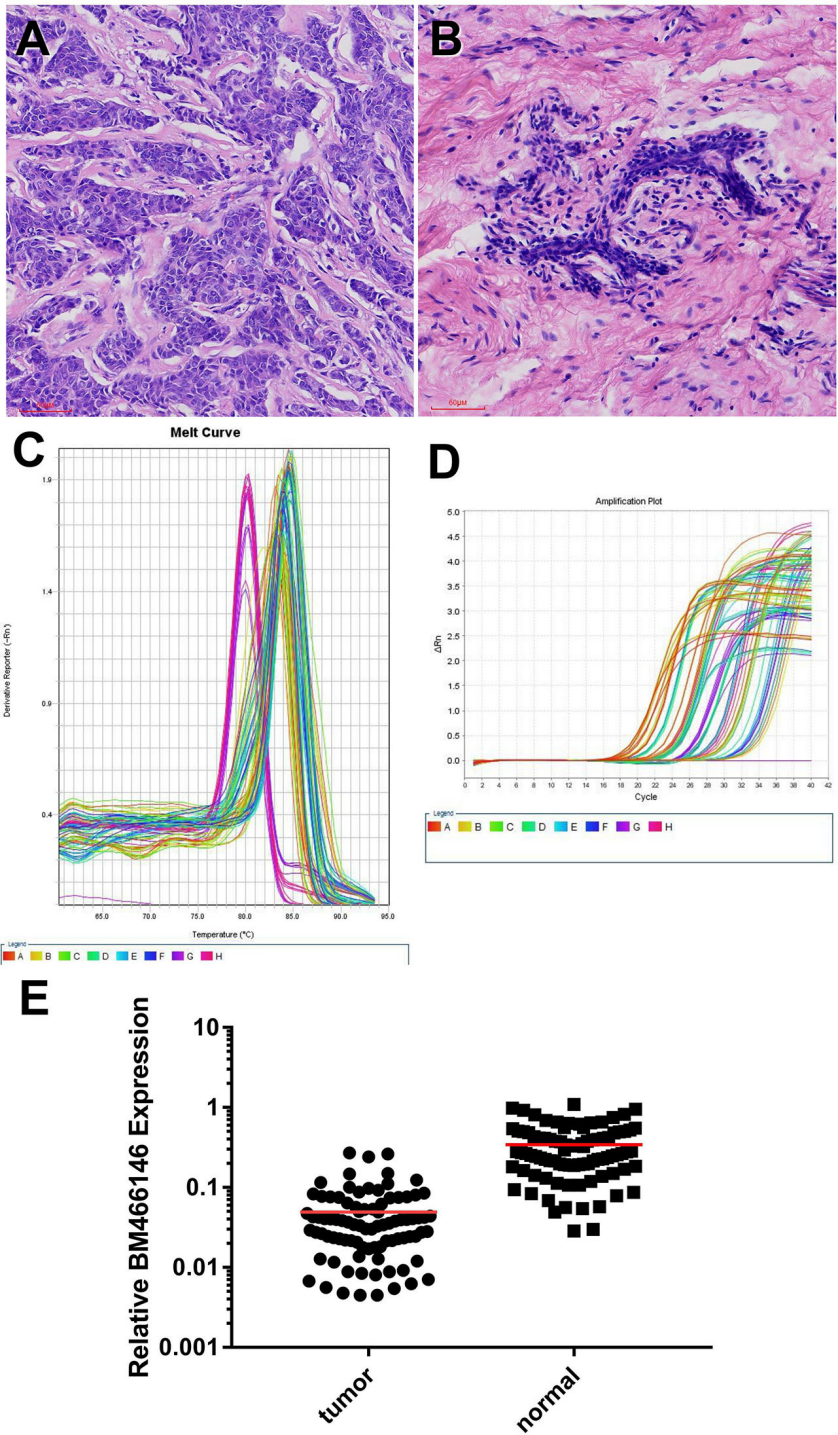
Expression of lncRNA BM466146 in breast cancer tissues and normal adjacent tissues **(A, B)** HE images of breast invasive carcinoma and normal breast tissues (HE×200). **(C, D)** RT-PCR results showed that the melting curves were all single peaks, no abnormal peaks, and no non-specific amplification. The four phases of the amplification curve at baseline phase, exponential growth phase, linear growth phase, and plateau phase are obvious. **(E)** The expression of lncRNA BM466146 is high in normal breast tissue and low in breast cancer tissue. The difference between the two is about 7.95 times (median change, *U*=385.000, *P*<0.001).

Data mining from microarray analysis of lncRNA in GEO (GSE72307) showed that the level of BM466146 was significantly lower in cancer compared to normal tissue. We collected 89 pairs of matched specimens from female patients with breast cancer, aged 31–82 years old, with an average age of 55 years. The size of the tumor is 0.8cm-9cm. The pathological types are invasive breast cancer (**Figures 1A, B**). The expression of BM466146 in these 89 pairs of breast cancer tissues and their adjacent tissues was evaluated by qRT-PCR. The specificity of the amplified product was verified by the gene dissolution curve. The experimental results showed that the lncRNA BM466146 is a single peak with good specificity with no non-specific amplification, and no abnormality in the amplification curve. The median expression of BM466146 in breast cancer tissues was 0.034236, and the median expression in normal breast tissues was 0.272265. The difference in the expression of lncRNA BM466146 between the two was 7.95 times. This result confirmed that the expression of BM466146 in breast cancer is significantly lower compared to normal breast tissues(*U*=385.000, *P*<0.001, **Figures 1C–E**).

### The Expression of lncRNA BM466146 Is Positively Correlated With CD8^+^T Cells and Negatively Correlated With Ki-67 Proliferation Index in Breast Cancer

**Figure 2:**
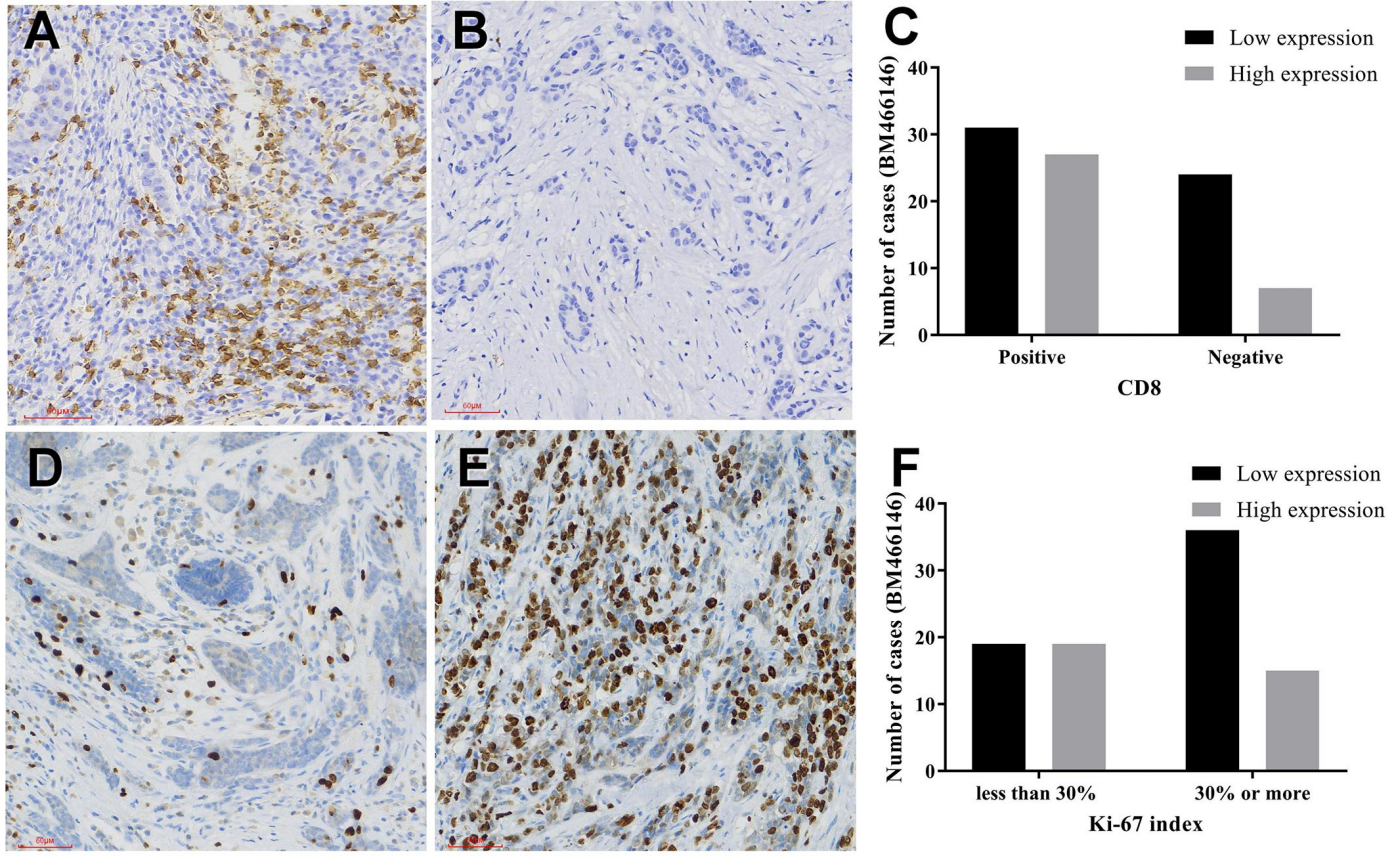
Immunohistochemical staining **(A, B)** CD8 immunohistochemical staining in breast cancer (**A** positive staining, 200x, **B** negative staining, 200x), **(C)** The relationship between BM466146 and CD8. **(D, E)** Immunohistochemical staining of Ki-67 (D Ki-67 index 5%,200x, E Ki-67 index 70%,200x), **(F)** the relationship between BM466146 and Ki-67 index.

Immunohistochemical staining for CD8 antibody was performed. Tumors with 10% or more positive CD8 staining were counted as positive staining, and less than 10% was negative staining. The cut off value of BM466146 expression in breast cancer was determined according to the ROC curve, and was 0.0411913. Based on this value, there were 34 cases of BM466146 high expression, and 55 cases of BM466146 low expression. In the 34 cases of BM466146 high expression group, 27 cases were CD8 positive and seven cases were negative. In the 55 cases of BM466146 low expression group, there were 31 CD8 positive samples and 24 negative samples. The number of CD8-positive T cells was different between the BM466146 high and the low expression group. The CD8 positive T cells was higher in the BM466146 high expression group, and the difference was statistically significant (*P*=0.027, *χ2* = 4.917, **Table 1**, **Figures 2A–C**). Spearman correlation analysis also confirmed that the number of CD8 positive cells was correlated with BM466146 expression (*P*=0.027, *r*=0.235).

**Table 1:** Relationship between BM466146 and Ki-67 and CD8.

	Number	BM466146		*χ2*	*P-value*
		Low expression	High expression		
CD8				4.917	0.027
Positive	58	31	27		
Negative	31	24	7		
Ki-67 index				3.910	0.048
<30%	38	19	19		
≥30%	51	36	15		

Cut-off a=0.05.

According to the Chinese Anti-Cancer Association Breast Cancer Diagnosis and Treatment Guidelines and Standards (2019 edition), 30% of Ki-67 index in breast cancer tissues is a critical value, with 30% or more as a high proliferation index, and less than 30% as a low proliferation index. Immunohistochemistry with Ki-67 antibody was performed. The Ki-67 index of breast cancer cells in BM466146 high expression group is different from that of low expression group. In the 34 cases of BM466146 high expression group, there was 44% cases with high Ki-67 proliferation index and 56% cases had low proliferation index. In the 55 cases of BM466146 low expression group, Ki-67 high proliferation index accounted for 65%, and low proliferation index accounted for 35%. These results indicated that the BM466146 low expression group had a high Ki-67 index and the difference was statistically significant (*P*=0.048, *χ2* = 3.910, **Table 1**, **Figures 2D–F**). Spearman correlation test results show that the expression of BM466146 is negatively correlated with Ki-67 index (*P*=0.049, *r* = -0.210).

### Overexpression of BM466146 Reduces the Proliferation of Breast Cancer Cells In Vitro

**Figure 3:**
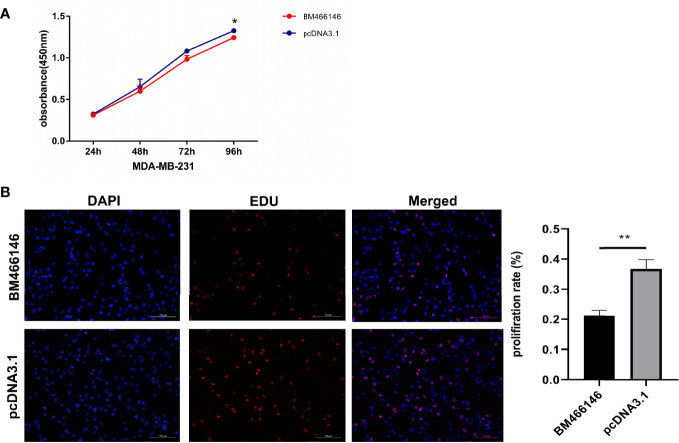
Overexpression of BM466146 inhibits breast cancer cell proliferation *in vitro*. **(A)** CCK-8 assay showed overexpression of BM466146 inhibits the proliferation of MDA-MD-231 cells (**P = 0.048); **(B)** EDU analysis showed that overexpression of BM466146 reduces the proliferation of MDA-MD-231 cells. The rate difference is 16% (P=0.0067).

CCK-8 experiment results showed that overexpression of BM466146 inhibited the proliferation of MDA-MD-231 cells (*P* = 0.048, **Figure 3A**), and the EDU experiment also confirmed that, compared with the control group, overexpression of BM466146 significantly reduced the proliferation of MDA-MD-231 cells with a difference of 16% (*P*=0.0067, **Figure 3B**). Transwell cell migration and invasion experiments confirmed that overexpression of BM466146 has no significant effect on the migration and invasion of MDA-MD-231 cells (**Supplementary 1**).

### Patients With High Expression of BM466146 Have Better Overall Survival

**Figure 4:**
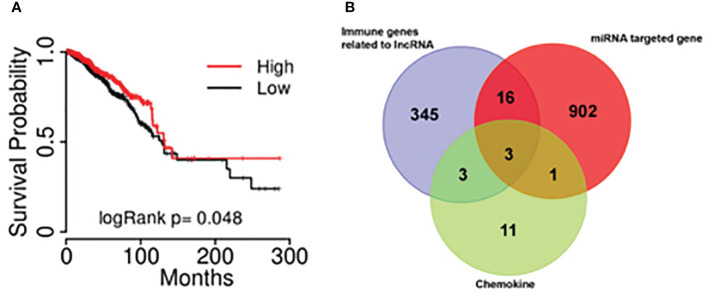
The results of bioinformatics **(A)** The association of lncRNA BM466146 expression and the prognosis of breast cancer patients. The prognosis of patients with high expression of BM466146 is better than that of patients with low expression, *P*=0.048; **(B)** Three genes (MIP1A, IL8, and CXCL-13) were in the intersection of lncRNA-related immune genes, MiRNA target genes, and CD8^+^T cell-related chemokines genes.

Kaplan-Meier survival analysis was performed using the combine data from the database of TCGA and hg19 gene and a survival curve was drawn (**Figure 4A**). The results showed that the expression of lncRNA BM466146 is positively associated with the prognosis of patients. Patients with high expression of BM466146 have better prognosis. Compared with patients with low expression of BM466146, patients with high expression of BM466146 have a significantly longer survival time, and the difference is statistically significant (*P*=0.048).

### Bioinformatics

Bioinformatics analysis was performed. A total of 19 miRNAs related to the BM466146 gene, and a total of 922 target genes of these miRNAs were found through chromosome colocalization and sequence identity matching. 373 immune function regulating genes related to lncRNA were retrieved through the ENCORI database, and there were 18 chemokines related to CD8^+^T cells. The three genes MIP1A, IL8 and CXCL13 were obtained in the intersection of the target gene of miRNA, the immune gene related to lncRNA and the chemokine related to CD8^+^T cell (**Figure 4B**). The CXCL-13 was chosen for further study after consulting with literatures (11, 12).

### The Relationship Between CXCL-13 and CD8

**Figure 5:**
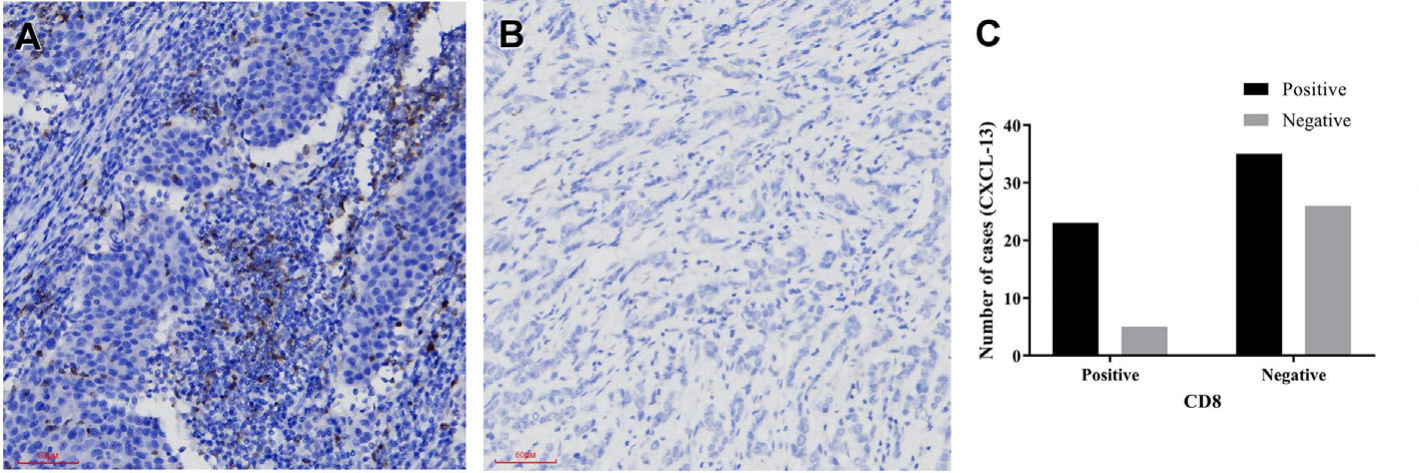
The relationship between CXCL-13 and CD8 **(A, B)** CXCL-13 immunohistochemical staining in breast cancer (**A** positive staining, 200x, **B** negative staining, 200x), **(C)** The relationship between CXCL-13 and CD8.

Immunohistochemistry was performed in these breast cancer and normal tissue specimens. In the tumors with CXCL-13 positive staining, 23 samples were CD8 positive and 35 samples were CD8 negative. In the tumors with CXCL13-negative staining, five samples were CD8 positive and 26 samples were CD8 negative. After statistical analysis, there are differences in the expression of CD8 between the CXCL-13 positive group and the negative group. The positive rate of CD8 in the CXCL-13 positive group was significantly higher than that in the CXCL-13 negative group, and the difference was statistically significant (*χ2* = 5.186, *P*=0.023, **Table 2**, **Figure 5**). The results of Spearman correlation analysis showed that expression of CXCL-13 was positively correlated with the number of CD8 positive cells (*P*=0.023, *r*=0.241).

**Table 2:** The relationship between CXCL-13 and CD8.

	Number	CXCL-13		χ2	*P-value*
		Positive	Negative		
CD8				5.186	0.023
Positive	58	23	35		
Negative	31	5	26		

Cut-off a=0.05.

## Discussion

Long non-coding RNA (lncRNA) is a type of regulatory RNA with a length of more than 200 nucleotides. It contains very few introns and open reading frames, and is not capable of encoding proteins. Most of them are transcription products of RNA polymerase II (13). According to the type and mode of action, it can be divided into five types: sense lncRNA, antisense lncRNA, bidirectional lncRNA, intergenic lncRNA and intragenic lncRNA (14). LncRNA is mostly located in the nucleus, and can be used as scaffold molecules, guide molecules, enhancers and molecular baits to participate in epigenetic modification, transcription and regulation (15–17). Studies have shown that lncRNA LINC00511 can promote the initiation of breast cancer (18), and lncRNA TINCR can promote epithelial-mesenchymal transition and resistance to trastuzumab in breast cancer (19). With the development of high-throughput sequencing, more and more lncRNAs have been identified playing roles in breast cancer, but their specific roles and mechanisms remain unclear. Currently, there was no specific lncRNA identified to be treatment target of breast cancer.

BM466146 is an intergenic lncRNA located on chromosome 18 and functions mainly in the nucleus. We found that the expression of BM466146 in normal breast tissue is much higher than that in breast cancer tissue. The results of survival analysis showed that patients with high expression of the BM466146 gene is associated with a better prognosis, and have a longer overall survival, which indicating that BM466146 has the effect of tumor suppressor genes. Simultaneously, statistical analysis found that in breast cancer, the Ki-67 index is low in patients with BM466146 high expression compared with those with low expression, suggesting that high expression of BM466146 can inhibit the proliferation of breast cancer cells. In vitro experiments also showed that BM466146 significantly inhibited the proliferation of breast cancer cells. It is well known that low Ki-67 index is associated with a good prognosis of breast cancer patients (20). Therefore, our study suggests that the high expression of lncRNA BM466146 in tumor tissues might predict improving prognosis of breast cancer.

It was known that lncRNA plays a vital role in mammalian epigenetics and transcriptional regulation, and is a key regulator of immune cell gene expression. It can control the number and homeostasis of immune cells in non-specific and specific immunity (21). The expression of certain lncRNA is associated with the development, differentiation and activation of immune cells (22). Studies have shown that lncRNA CNC-TIM3 exacerbates CD8 T cell failure by binding to TIM-3 in hepatocellular carcinoma (23), and down-regulating lncRNA NEAT1 can inhibit apoptosis of CD8^+^ T cells and increase the anti-tumor activity of CD8^+^ T cells in hepatocellular carcinoma (24). Therefore, lncRNA can regulate the activation, proliferation, apoptosis, and anti-tumor activity of CD8^+^ T cells.

Cytotoxic T Lymphocytes (CTL) are the first line of cancer-targeted immune cells. Tumor-associated fibroblasts, M2 macrophages and regulatory T cells can produce an immune barrier against CD8^+^ T cells in the process of tumor development. In anti-tumor immunity, CD8^+^ T cells are activated and proliferated, and their anti-tumor function increased to produce a durable and effective anti-tumor immune response (25).

lncRNA represents the largest class of noncoding RNAs (ncRNAs) which are mainly involved in gene regulation. In addition to transcriptional regulation, lncRNA also regulates mRNA splicing, inhibits translation, and plays a role in mRNA processing, maturation and stability (26). MiRNAs are small ncRNAs with a length of about 22 nucleotides. By binding to specific sites of mRNA and miRNA response elements (MRES), they perform post-transcriptional regulation, leading to transcription degradation or translation inhibition. LncRNAs can competitively bind to miRNAs with the same MRES, dilute the concentration of free miRNAs in cells, reduce the inhibition of miRNA on mRNA, and thus increase the expression of downstream genes. This phenomenon is known as the competing endogenous RNA (ceRNA) hypothesis (27). lncRNA can regulate the chemotaxis and activation of CD8^+^ T cells by regulating the expression of mRNA, but it cannot directly act on CD8^+^ T cells.

Based on the analysis of biogenesis in the TCGA database, the crossover of BM466146-related miRNA target genes, lncrNA-related immune genes and CD8^+^ T-cell-related chemokines was performed. And it was found that lncRNA BM466146 may be used as ceRNA to adsorb HSA-Mir-224-3P, up-regulate the expression of CXCL-13 gene, and then generate more CXcl-13 proteins to activate CD8^+^T cells and play its cytotoxic role. CXCL-13 protein is also known as B lymphocyte chemokine 1 (BCA1), a small cytokine belonging to the CXC chemokine family. This chemokine has a selective chemotactic effect on B cells belonging to the B-1 and B-2 subsets, and stimulates its effect by interacting with the only chemokine receptor CXCR5 (11). CD8 ^+^ CXCR5 ^+^ T cells are a subset of CD8 ^+^ T cells, which have potential cytotoxic effects in chronic viral infections and cancers (28, 29). This study found that the number of CD8^+^ T cells increased in the breast cancer that overexpressed BM466146. The proportion of CD8^+^ T cells in the CXCL-13 positive group was significantly higher than that in the CXCL-13 negative group. In breast cancer, BM466146 might be able to up-regulate the expression of CXCL- 13 gene, which in turn activates CD8 ^+^ T cells in the tumor microenvironment and plays a tumor suppressor effect. This result is in consistent with the results of Bai et al. in pancreatic cancer (12). CD8 ^+^ T cells in the tumor microenvironment have higher cytotoxicity than CD8 ^-^ T cells and indicate a better prognosis.

In summary, this study found that lncRNA BM466146 has the function of a tumor suppressor gene. The high expression of lncRNA BM466146 is associated with better prognosis. BM466146 could promote the expression of CXCL-13 gene and increase the number of CD8^+^ T cells in tumor microenvironment, and activate their recognition and killing effect on tumor cells.

## Data Availability Statement

The original contributions presented in the study are included in the article/**Supplementary Material**. Further inquiries can be directed to the corresponding author.

## Ethics Statement

The studies involving human participants were reviewed and approved by Ethics Committee of Weifang People’s Hospital. The patients/participants provided their written informed consent to participate in this study.

## Author Contributions

YZ and PG conceived and designed the study. YZ, XD, YF, and GX performed experiments and analyzed results. GH, LJ, YW, and LW collected clinical samples and analyzed results. DY, JZ, and XG conducted experiments and organized data. YZ wrote the first draft. PG edited the article. All authors contributed to the article and approved the submitted version.

## Funding

This study was supported by the National Natural Science Foundation of China (grant nos. 81372856 and 81672842) and the Taishan Scholars Program of Shandong Province (grant no. ts201511096).

## Conflict of Interest

Author DY was employed by the company Geneis Beijing Co., Ltd.

The remaining authors declare that the research was conducted in the absence of any commercial or financial relationships that could be construed as a potential conflict of interest.
