# Supplementary material for: Overexpression of LncRNA BM466146 Predicts Better Prognosis of Breast Cancer
Source: Front Oncol. 2021 Jan 29;10:628757. doi: 10.3389/fonc.2020.628757 (PMC7878538; doi:10.3389/fonc.2020.628757)
Supplement: Supplementary file 1 [file DataSheet_1.docx]

Cell migration and invasion experiments


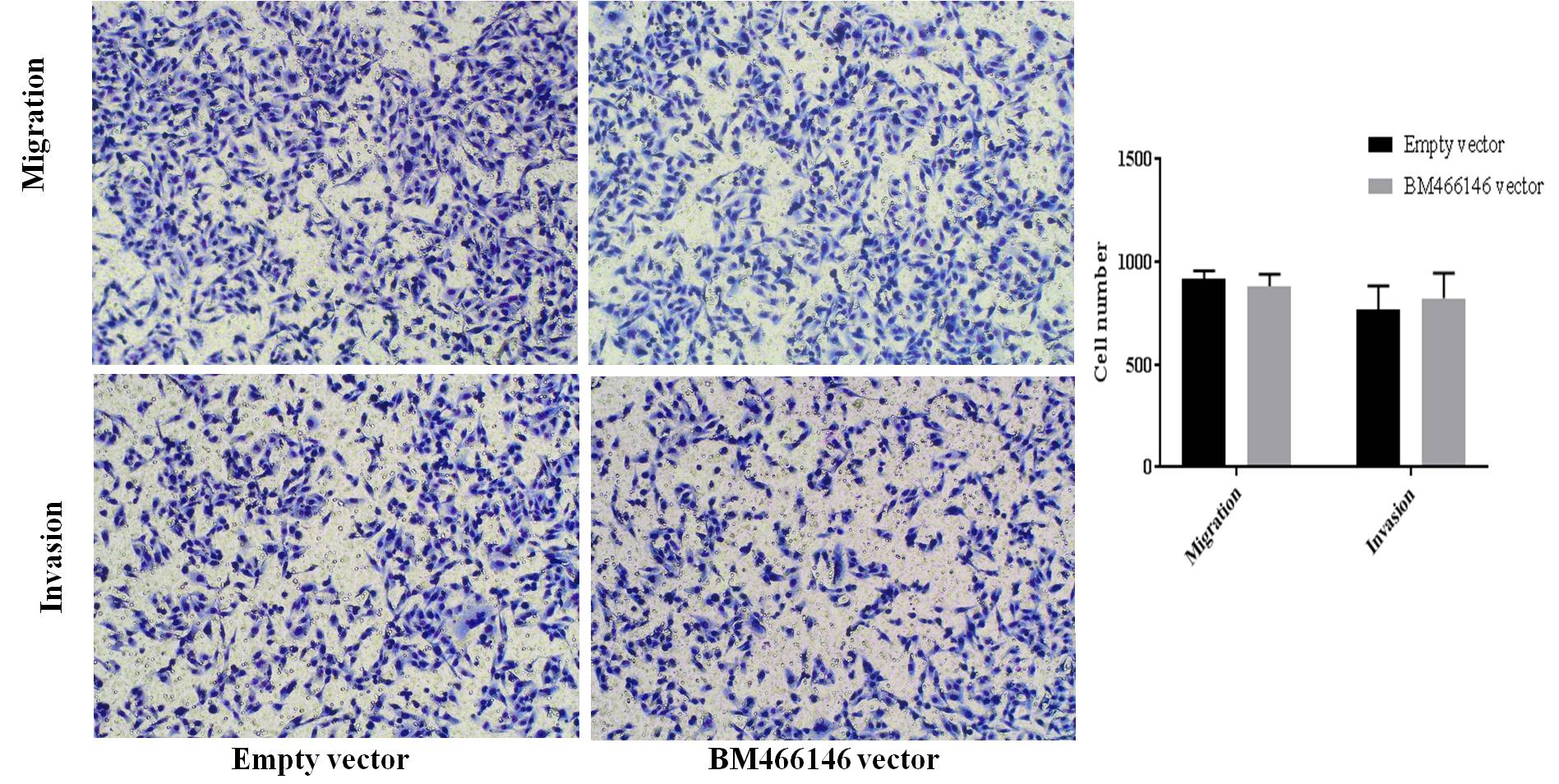


The results of cell migration and invasion experiments showed that overexpression of BM466146 did not inhibit the migration and invasion of MDA-MD-231cells (migration *P*=0.60, invasion *P*=0.68).
